# A Novel, Drinkable Food Supplement Formulation Reduces Hair Shedding and Increases the Percentage of Anagen Scalp Hair Follicles in Females with Hair Loss

**DOI:** 10.3390/jcm14238471

**Published:** 2025-11-28

**Authors:** Manuel Sáez Moya, Gillian E. Westgate, Ralf Paus, Daniela Grohmann

**Affiliations:** 1 Olistic Research Labs S.L., 08021 Barcelona, Spain; 2 Gill Westgate Consultancy Ltd., Stevington MK43 7QT, UK; 3 Dr. Phillip Frost Department of Dermatology & Cutaneous Surgery, University of Miami Miller School of Medicine, Miami, FL 33125, USA; 4 CUTANEON—Skin & Hair Innovations GmbH, 22335 Hamburg, Germany

**Keywords:** nutraceutical, oral supplementation, hair growth, hair follicle cycling, women

## Abstract

**Background/Objectives**: Telogen effluvium (TE) is a common, non-scarring hair loss condition characterized by excessive shedding due to disruptions in the hair growth cycle. It is often triggered by stress, hormonal changes, or nutritional deficiencies and is often associated with impaired quality of life. The objective of this study was to evaluate the efficacy and safety of a novel once-a-day drinkable food supplement in women experiencing TE. **Methods**: A monocentric, open-label, single-arm pilot study was conducted, enrolling 37 female subjects aged 20 to 45 years with self-perceived hair shedding and diagnosed with TE. Subjects refrained from using products with similar effects throughout the study. Evaluations included hair density, hair shedding, anagen to catagen/telogen (A:C/T) ratio, and self-perception after 1, 3, and 6 months. Statistical analyses were performed using Linear Mixed-effects Models (LMMs) and Wilcoxon signed-rank tests. **Results**: At 1, 3, and 6 months, a statistically significant increase in hair density compared to baseline was observed under the regimen of the tested product. After 6 months, this translated into a 12% increase vs. baseline (*p* < 0.001). Hair shedding decreased significantly from baseline to each subsequent visit, with a 28% reduction in shedding after 6 months (*p* < 0.05). The A:C/T ratio significantly increased after both 3 and 6 months, from 3.39:1 to 6.96:1 (*p* < 0.001). Self-perception questionnaires indicated high satisfaction with hair improvements. **Conclusions**: This single-arm pilot study suggests that the novel, drinkable food supplement improves hair density and hair shedding in women experiencing TE and underscores the potential of supplement intervention for managing female hair thinning, mainly by reducing TE through increased density of growing hairs. Whilst these preliminary results are encouraging, we recognize that a larger, placebo-controlled, blinded, randomized trial using the product is necessary to corroborate these findings and further explore the underlying hair cycle effects.

## 1. Introduction

Female hair loss and overall hair thinning are conditions that arise due to alteration of the hair growth cycle, namely shortening of the growth phase of the hair cycle (anagen) and premature hair follicle (HF) regression (catagen), leading clinically to TE [1]. TE is the most frequent form of hair loss in women, and can have multiple different causes, ranging from medication, nutritional deficiencies, hormonal imbalances or virally induced TE to female pattern hair loss (FPHL), with which TE is most often associated. In FPHL, TE is typically also associated with some HF miniaturization [2,3,4,5]. Both the degree of hair loss and its associated psychological effects are often underestimated in females, yet FPHL affects women nearly as commonly as pattern hair loss in men, with estimates of at least 50% hair loss in women by age 50, compared to 40% by age 35 in men, reaching up to 70% in later decades of life in men [5,6,7]. Early-stage FPHL, which often presents initially with TE, is probably under-diagnosed [8]. Acute or chronic hair shedding, follicle miniaturization and overall hair thinning, may greatly impair self-esteem and self-confidence, causing psychological problems, and is often associated with reduced quality of life [9,10].

In recent years, food supplements, medicinal plants and functional foods have attracted increasing attention, and the influence of diet on hair health is more frequently being considered in consultation with a dermatologist or trichologist [11,12,13,14,15,16,17]. The excellent vascularization and high perfusion rate of scalp HFs and the fact that these serve as very effective excretory organs which specialize in taking up and enriching a multitude of serum components, in principle, make scalp HFs excellent targets for supplement interventions [18,19,20]. However, evidence that such interventions are effective in clinical reality is limited, with no prior evidence of benefits from a drinkable food supplement formulated for hair loss [21,22]. It is presently unclear to what extent TE in association with FPHL is associated with micronutrient deficiencies, the correction of which could theoretically benefit affected individuals. Data on nutrient deficiency is often low quality or case specific [23].

However, a recent metabolic analysis, which compared intermediate (shorter/smaller) with terminal scalp HFs in women affected by FPHL, has shown that selected essential amino acids and vitamins are indeed decreased in intermediate HFs [24], i.e., those HFs which are in the process of being miniaturized. Moreover, these HFs exhibit a more quiescent metabolic phenotype, while nutrient uptake, vascularization, and angiogenesis-associated growth factor expression, as such, do not seem to be impaired in organ-cultured female scalp HFs [24]. Also, many food ingredients have already been documented to prolong anagen and/or improve other hair growth parameters in human scalp HFs ex vivo [25,26,27,28]. This more systematic exploration of nutritional approaches to hair loss management with an evidence-based medicine approach in clinical hair loss studies [17], builds upon the growing understanding of hair physiology, the control of the hair cycle, and the many-faceted non-pharmacological routes to improving hair health where data from topical products has been generated [12,29].

A novel oral food supplement in liquid form for daily intake has been rationally designed to target multiple pathways that may help manage hair loss in women. This food supplement presents six distinct categories of active ingredients, including more than 30 components in its formula. The formula includes plant extracts with clinically proven 5-α reductase-inhibitory activity (e.g., *Serenoa repens*, *Prunus Africana*, and *Cucurbita pepo*) [30,31], antioxidants (e.g., Vitamin E, Resveratrol, and Astaxanthin) [32,33], anti-inflammatory ingredients (e.g., *Curcuma longa* and *Nigella sativa*) [34], and an adaptogen (Ashwagandha), a natural compound considered to facilitate stress adaptation responses [35], along with amino acids known to be keratin precursors, such as L-Cysteine and L-Arginine [36,37,38], as well as vitamins and minerals known to play crucial roles in cell proliferation and/or hair shaft production, such as Vitamin A, Biotin (vitamin B7/H), and iron [24,39,40,41].

As a first step towards an evidence-based approach to clinical hair loss studies, in this initial, hypothesis-generating clinical pilot study, we aimed to evaluate the efficacy and safety of this drinkable food supplement in a population of women who self-reported hair shedding problems, including TE and/or FPHL, using an open-label, monocentric, single-arm study [17].

## 2. Materials and Methods

### 2.1. Study Design and Population

37 female Caucasian subjects were enrolled in this monocentric, open-label, single-arm pilot study, with 33 finishing the study; four dropouts did not complete attendance at the required appointments. No dropouts due to volunteer-reported adverse effects were seen. The study data included a cohort of 33 female subjects, with ages ranging from 20 to 45 years (mean = 32.05; standard deviation (SD) = 8.16), that completed the study (Table 1). Each subject was informed verbally and in writing about the characteristics of the study, before providing informed written consent. The study was carried out under the Good Clinical Practices (Guideline for good clinical practice E6 (R2) of 14 June 2017, EMA/CHMP/ICH/135/1995 of 1 May 1996, European Parliament, and Council Guideline 2001/20/CE—1 May 2001).

In this study, the subject cohort comprised healthy female subjects who were recruited based on having self-perceived increased hair shedding to such an extent that they would seek dermatological consultation. The board-certified dermatologist study investigator subsequently included subjects either diagnosed with TE (29) by visual identification and pull test or with FPHL (4) based on accepted diagnostic criteria [8]. All subjects committed to refraining from the use of systemic, topical, or oral products with hair growth claims that might exert similar effects to the test product throughout the study period.

Exclusion criteria included pregnancy, post-partum (6 months) or nursing women; skin or scalp disorders such as psoriasis and dermatitis; scarring alopecia and alopecia areata; allergy or hypersensitivity to any compound of the test product or any other declared allergy or food intolerance; a self-disclosed medical history encompassing systemic diseases such as hepatic or renal impairment, cardiac anomalies, neurological, or psychological ailments; or drug treatments like anticoagulants, antidepressants, or minoxidil. A list of all inclusion and exclusion criteria is presented in Table 2.

Before inclusion, subjects signed an informed consent form expressing their understanding of the investigational nature and procedure of the study. This open-label, single-arm study was designed as a preliminary proof-of-concept pilot trial preceding and to inform a double-blind, placebo-controlled study to be conducted in the future. Consequently, ethics committee approval was not sought, given its nutritional and pilot nature, nor was the study listed in the EU clinical trials register. Nevertheless, ethical guidelines and good clinical practices for human research were rigorously followed throughout the study.

### 2.2. Test Product

All subjects received the test product. This food supplement is presented in a drinkable format, allowing the intake of over 30 natural ingredients in a daily dosage of one 25 mL vial. A list of all active ingredients can be found in Table S1. All subjects were asked to self-administer the test product once daily after breakfast or lunch between January and July, i.e., within a standardized period of the year. This is important since seasonal fluctuations in hair loss are well-documented [42,43,44].

### 2.3. Study Endpoints and Procedures

Evaluations were performed by expert evaluators from Zurko-CTC (Zurko-Centro de Tecnología Capilar, Barcelona, Spain) at the baseline visit (T0), and after one month (T1), three months (T2), and six months (T3) after taking the test product.

The primary endpoint was the evaluation of the A:C/T (anagen to catagen/telogen) ratio carried out through a pluck trichogram, where 25–30 scalp hairs were extracted by traction to examine their hair roots under the microscope and evaluate in which phase of the hair growth cycle they were in (anagen—growth phase; or catagen/telogen—regression/quiescence phase) [45]. A representative microscope image illustrating the differences between anagen and catagen/telogen hairs is shown in Figure S1.

The secondary endpoints were evaluations of hair density and hair shedding and the subjects’ responses from the self-perception questionnaire. Hair density (no. of hairs/cm^2^) was assessed on the parietotemporal area of the head by phototrichogram. Hair in this section of the scalp was clipped uniformly short and an image of this area of scalp including the evaluation area was captured. Subsequently, the same area was re-photographed after 72 h and the same procedure was followed in the other visits (having the first image as a reference to capture the same area), facilitated by the observable presence of shorter hair in the parietotemporal region. Microscopic images with 20× magnification were captured and analyzed with TrichoScan HD 4.0 and TrichoScan^®^ software V3.6.44.146 (DermoScan GmbH, Regensburg, Germany) [46,47]. Hair shedding was evaluated through a standardized combing and washing test [45,48,49,50]. All shed hairs were collected for subsequent counting. Subjects attended visits T0 and T3 without having washed their hair for a minimum of 48 h and without combing for at least 24 h prior to the visit. A self-perception questionnaire was filled out by all subjects at T1, T2, and T3 to evaluate their perception on the effects of the test product (e.g., hair density, hair shedding, hair volume, and product ease of use). All questions used a 6-point Likert scale with the following responses: strongly agree, agree, slightly agree, slightly disagree, disagree, and strongly disagree. Throughout all study phases, the adherence of volunteers to the protocol was assessed by counting the number of empty vials that the volunteers brought to each control visit.

The safety endpoints included the identification and assessment of potential adverse events (AEs) by a dermatologist from Zurko-CTC.

### 2.4. Statistical Analysis

Data characterization was performed for the clinical data at different experimental times (T0 to T3), including mean, SD, standard error of mean (SEM), and percentage values. The efficacy of the test product was expressed in absolute values (mean) versus baseline (evaluation of tested product vs. T0). For normal distribution of clinical data, a Linear Mixed-effects Model (LMM) was used to compare results at each visit versus baseline. The Wilcoxon signed-rank test was used when data followed a non-normal distribution. The paired Student’s *t*-test was used to analyze differences between two timepoints, namely data from T0 compared to T3. *p*-values less than 0.05 were considered statistically significant.

## 3. Results

### 3.1. The Tested Drinkable Food Supplement Significantly Increases the Percentage of Hair Follicles in the Anagen Phase

**Figure 1:**
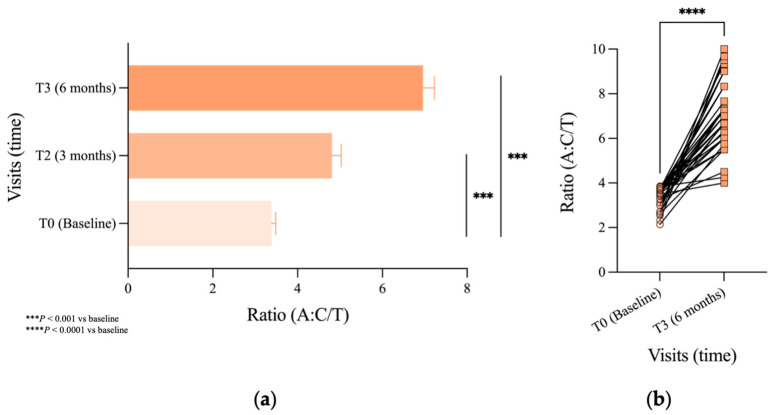
Evaluation of A:C/T ratio variation via pluck trichogram. (**a**) A:C/T ratio from baseline (T0) to visits T2 and T3 was statistically analyzed with the LMM (SEM is represented for all time visits: T0:0.09; T2:0.22; T3:0.27); (**b**) A:C/T ratio from baseline to visit T3 statistically analyzed with Student’s paired *t*-test. Significance: *** *p* < 0.001 vs. T0; **** *p* < 0.0001 vs. T0.

A statistically significant difference in the A:C/T ratio was observed already after 3 months (T2), showing an increase from 3.39:1 to 4.81:1 (42%; *p* < 0.001), followed by an increase to 6.96:1 (105% compared to T0; *p* < 0.001) after 6 months (T3) (see Figure 1a). Independent analysis by paired Student’s *t*-test confirmed a highly significant increase in the percentage of anagen over telogen HF after 6 months (*p* < 0.0001), as shown in Figure 1b.

### 3.2. The Tested Food Supplement Increases Hair Density over Time

**Figure 2:**
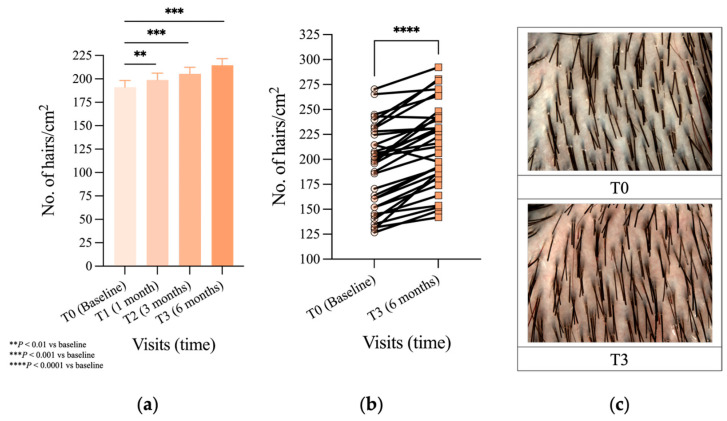
Quantitative analysis of hair density (hairs/cm^2^) via TrichoScan^®^ phototrichogram. (**a**) Absolute number of hairs per cm^2^ over 6 months statistically analyzed with the LMM (SEM is represented for all time visits: T0:7.23; T1:7.26; T2:6.90; T3:7.18); (**b**) absolute number of hairs per cm^2^ from baseline to visit T3 statistically analyzed with Student’s paired *t*-test; (**c**) Representative examples of phototrichogram at visits T0 vs. T3. Significance: ** *p* < 0.01 vs. T0; *** *p* < 0.001 vs. T0; **** *p* < 0.0001 vs. T0.

Total hair density (vellus and terminal hairs included) showed a statistically significant increase in the absolute average hair density at T1 of 7.78 hairs/cm^2^ (4%, *p* < 0.01) when compared to baseline. Furthermore, at three (T2) and six (T3) months hair density continued to progressively increase with an additional 14.28 hairs/cm^2^ (8%, *p* < 0.001) and 23.39 hairs/cm^2^ (12%, *p* < 0.001) (see Figure 2a and Table 3). When hair density data were compared between T0 and T3 separately through a paired Student’s *t*-test, this confirmed the increase to be highly statistically significant (*p* < 0.0001), as shown in Figure 2b, and illustrative phototrichogram images from a representative subject at these time points are displayed in Figure 2c.

### 3.3. Self-Perception Questionnaire Data Reflect Perceived Hair Health Benefits

Results from the self-assessment questionnaire were consistent with the quantitative endpoints. As summarized in Table 4, at the end of the study (6 months—T3), subjects reported perceiving their hair healthier, stronger, and shinier (94%). They also reported a hair volume increase (85%) as well as reduced hair shedding (91%). Overall, their satisfaction with the re-densifying effects of the test product was notably high (97%).

### 3.4. Hair Shedding Significantly Decreases After 6 Months, Indicating Reduced TE

**Figure 3:**
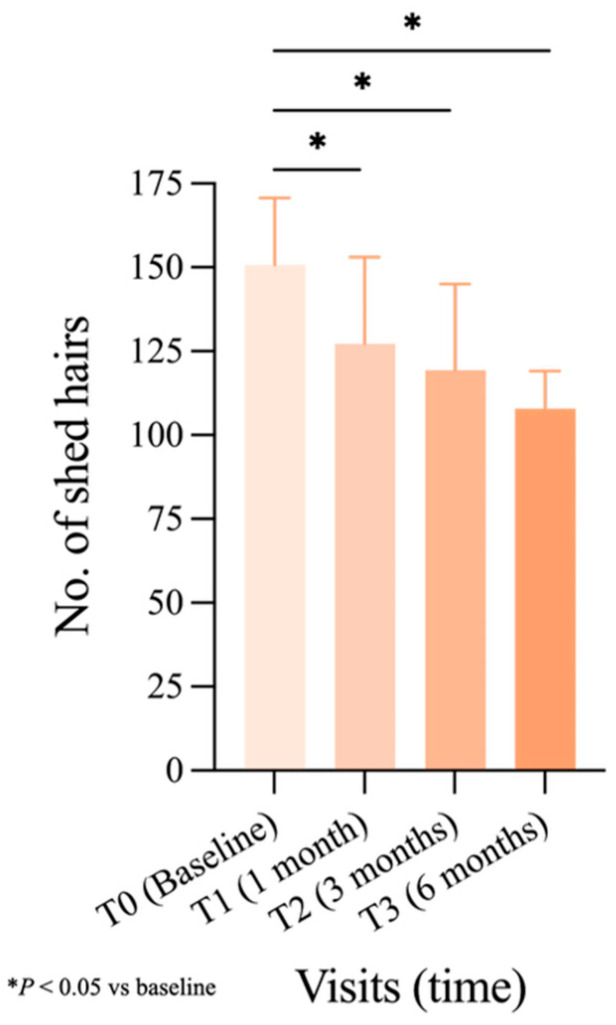
Change in the number of collected hairs (through Combing + Washing Test) at each time visit, statistically analyzed with the Wilcoxon signed-rank test (SEM is represented for all time visits: T0:20.04; T1:25.77; T2:25.77; T3:11.22). Significance: * *p* < 0.05 vs. T0.

In Figure 3, the total number of shed hairs (Combing + Washing test) from T0 to T3 is summarized, data was analyzed by Wilcoxon signed-rank test, showing statistically significant differences between baseline and each visit. A significant (*p* < 0.05) decrease compared to baseline (150.70 shed hairs) appears at T1 (127.27 shed hairs (−16%)), persisting at T2 and T3, with a progressive decrease to 119.30 shed hairs (−21%) after 3 months and reaching 107.85 shed hairs (−28%) after 6 months.

### 3.5. Safety

No adverse effects associated with the test product were observed or reported throughout the study and the subjects’ self-assessment questionnaire confirmed that the test product was well-tolerated.

## 4. Discussion

This current 6-month pilot study is the first to explore whether a daily drinkable supplement formulation impacts significantly on A:C/T ratio, hair density, shedding, and self-perceived benefits in women experiencing hair shedding who had been diagnosed with TE by a dermatologist. Following previous study designs, the methods employed included phototrichogram through TrichoScan^®^ analysis, standardized combing and washing tests, and pluck trichogram, as well as evaluation of a self-assessment questionnaire [50,51,52].

Study limitations are the relatively low number of subjects, and its open, single-armed design. The absence of a placebo control group in this initial, exploratory pilot trial was motivated by the challenge to generate a drinkable placebo product that tastes, looks, and smells exactly like the test supplement, but does not contain any hair growth-modulatory ingredients. Generating such a placebo control will be critical to a planned follow-up study, not the least given the recently appreciated role that chemosensory receptors have in human hair growth regulation [27,53,54]. However, given that seasonal fluctuations in HF cycling and hair growth are well-appreciated, it should be emphasized that the current pilot study examined all subjects within the same seasonal window [43,44]. Moreover, subjects were not stratified into specific TE subtypes within the study population; nevertheless, based on the demographic and clinical characteristics assessed, the nutraceutical demonstrated efficacy across the enrolled cohort. In a follow-up study, the application of more rigorous definition and inclusion criteria for TE, e.g., following Harries et al. (2016) and Jimenez et al. (2021), as well as higher numbers, will be advisable to increase stratification of the enrolled subjects [8,54].

With these study limitations in mind, our preliminary results support that the tested food supplement formulation significantly reduced hair shedding over time and increased both the A:C/T ratio and hair density. Taken together, these findings strongly support that the drinkable food supplement effectively reduced TE—likely by reducing the percentage of hair follicles entering catagen and telogen, either by prolonging the duration of anagen or by recruiting a greater proportion of telogen HFs back into anagen.

It is encouraging to note that these results were reflected in the subjects’ subjective perceptions of improved hair volume and hair quality, and self-perceived overall “hair health”. Notably, the current pilot study is the first of its kind to provide quantitative data that a daily drinkable supplement can improve hair density and reduce hair shedding in women experiencing TE. In a larger-scale optimally designed follow-up trial, it will now be important to demonstrate that these effects can be demonstrated against an optimal placebo product.

## 5. Conclusions

Findings from this single-arm proof-of-concept pilot study indicate that the innovative drinkable food supplement may improve hair density and reduce hair shedding in women affected by TE. These results highlight the potential role of nutraceuticals in addressing female hair loss, primarily by decreasing TE through an increase in the proportion of growing hairs. Although the outcomes are promising, confirmation through a larger, randomized, placebo-controlled, and blinded clinical study is warranted to validate these effects and to investigate the supplement’s specific influence on hair cycle dynamics.

## Figures and Tables

**Table 1:** Summary of baseline subject demographics.

	All (n = 33)
Mean age (SD), years	32.05 (8.16)
Age range, years	20–45
Ethnicity, n (%)	
Caucasian	33 (100%)
Hair type, n (%)	
Type 1	20 (60.6%)
Type 2	10 (30.3%)
Type 3	3 (9.1%)
Type 4	-

**Table 2:** Summary list of inclusion and exclusion criteria.

Inclusion Criteria	Exclusion Criteria
Aged 20–45 years Sex: female Self-perceived excess hair shedding, diagnosed with either TE or FPHL Otherwise healthy volunteers Commitment to not use systemic, topical or oral products with similar effects to the test product throughout the study Signing the informed consent form Availability to attend all control visits Commitment to maintain existing daily routine, including cosmetic and dietary habits	Known allergy or hypersensitivity to any of the ingredients of the test product or any other allergy or food intolerance Subjects that previously took the test product Subjects who had participated in similar studies or had used anti-hair loss products within the last 3 months Subjects with history of hair transplant Skin or scalp diseases/disorders (e.g., psoriasis, dermatitis, eczema, alopecia cicatricial or areata) Subjects that present alopecia as consequence of any medical disease (hypothyroidism, anemia, lupus, etc.) Subjects undergoing chronic drug treatment or diagnosed with a systemic disease Subjects who initiated a hormonal treatment (oral or topical contraceptives) within the 6 months preceding the study start Pregnant, post-partum (6 months), or nursing women Stressful incident within the last 6 months

**Table 3:** Hair density over 6 months.

	Absolute no. of Hairs (Average, SD)	LMM * p-Values
**T0**	191.13 (41.55)	-
**T1**	198.91 (41.72)	*p* < 0.01
**T2**	205.42 (39.65)	*p* < 0.001
**T3**	214.53 (41.26)	*p* < 0.001

* LMM was used to statistically analyze hair density data.

**Table 4:** Self-assessment questionnaire results after 6 months of taking the test product.

Percentage of Subjects (%) *	Self-Perception Results
94%	Healthier hair
94%	Stronger hair
85%	Overall hair volume
94%	Shinier hair
91%	Overall decrease in hair shedding
97%	Re-densifying effect satisfaction

* Percentage of subjects (from slightly agree to strongly agree) reporting improvement after 6 months with the test product.

## Data Availability

The dataset that supports the findings of this study is available on request from the corresponding author.

## Supplementary Materials

The following supporting information can be downloaded at: https://www.mdpi.com/article/10.3390/jcm14238471/s1, Figure S1: Representative microphotography of catagen and anagen hairs; Table S1: List of active ingredients of the tested product.

## Abbreviations

The following abbreviations are used in this manuscript:

A:C/T	Anagen to Catagen-Telogen ratio
FPHL	Female Pattern Hair Loss
HF	Hair Follicle
LMM	Linear Mixed-effects Model
