# Supplementary material for: A Novel, Drinkable Food Supplement Formulation Reduces Hair Shedding and Increases the Percentage of Anagen Scalp Hair Follicles in Females with Hair Loss
Source: J Clin Med. 2025 Nov 28;14(23):8471. doi: 10.3390/jcm14238471 (PMC12693284; doi:10.3390/jcm14238471)
Supplement: Supplementary file 1 [file jcm-14-08471-s001.zip › Table S1.pdf]

Table S1. List of active ingredients of the tested product.

**TEST PRODUCT ACTIVE INGREDIENT LIST**
Saw palmetto extract (*Serenoa repens*, fruit)
Pygeum extract (*Prunus africana*, bark)
Pumpkin seed extract (*Cucurbita pepo*, seeds)
Dunaliella salina extract, algae (7.5% β-carotene)
Folic acid (pteroylmonoglutamic acid)
L-ascorbic acid (vitamin C)
Cholecalicferol (vitamin D3)
Biotin (D-biotin)
Zinc bisglycinate
Micronized iron (ferric pyrophosphate)
L-selenomethionine
Fucus vesiculosus extract, algae (0.1% iodine)
Collagen precursor amino acids (L-Glycine, L-Proline, L-Hydroxyproline, L-Alanine, L-Serine, L-Leucine, L-Phenylalanine, L-Isoleucine)
Keratin precursor amino acids (L-Arginine, L-Lysine, L-Tyrosine)
L-Cysteine
L-Methionine
Hyaluronic acid (sodium hyaluronate)
Horsetail extract (*Equisetum arvense*, herb)
Millet extract (*Panicum miliaceum*, seed)
Tyndallized *Lactobacillus plantarum*
Tyndallized *Lactobacillus paracasei*
Tyndallized *Lactobacillus reuteri*
Cursol® turmeric extract (*Curcuma longa*, rhizome)
Black cumin extract (*Nigella sativa*, seeds)
Bilberry extract, >5% anthocyanidins (*Vaccinium myrtillus*, fruit)
DL-α tocopheryl acetate (vitamin E)
Polygonum cuspidatum extract (98% resveratrol)
Haematococcus pluvialis extract (astaxanthin)
Sensoril® ashwagandha extract (*Withania somnifera*, root & aereal parts)
