# Supplementary figures and images for: A Novel, Drinkable Food Supplement Formulation Reduces Hair Shedding and Increases the Percentage of Anagen Scalp Hair Follicles in Females with Hair Loss
Source: J Clin Med. 2025 Nov 28;14(23):8471. doi: 10.3390/jcm14238471 (PMC12693284; doi:10.3390/jcm14238471)

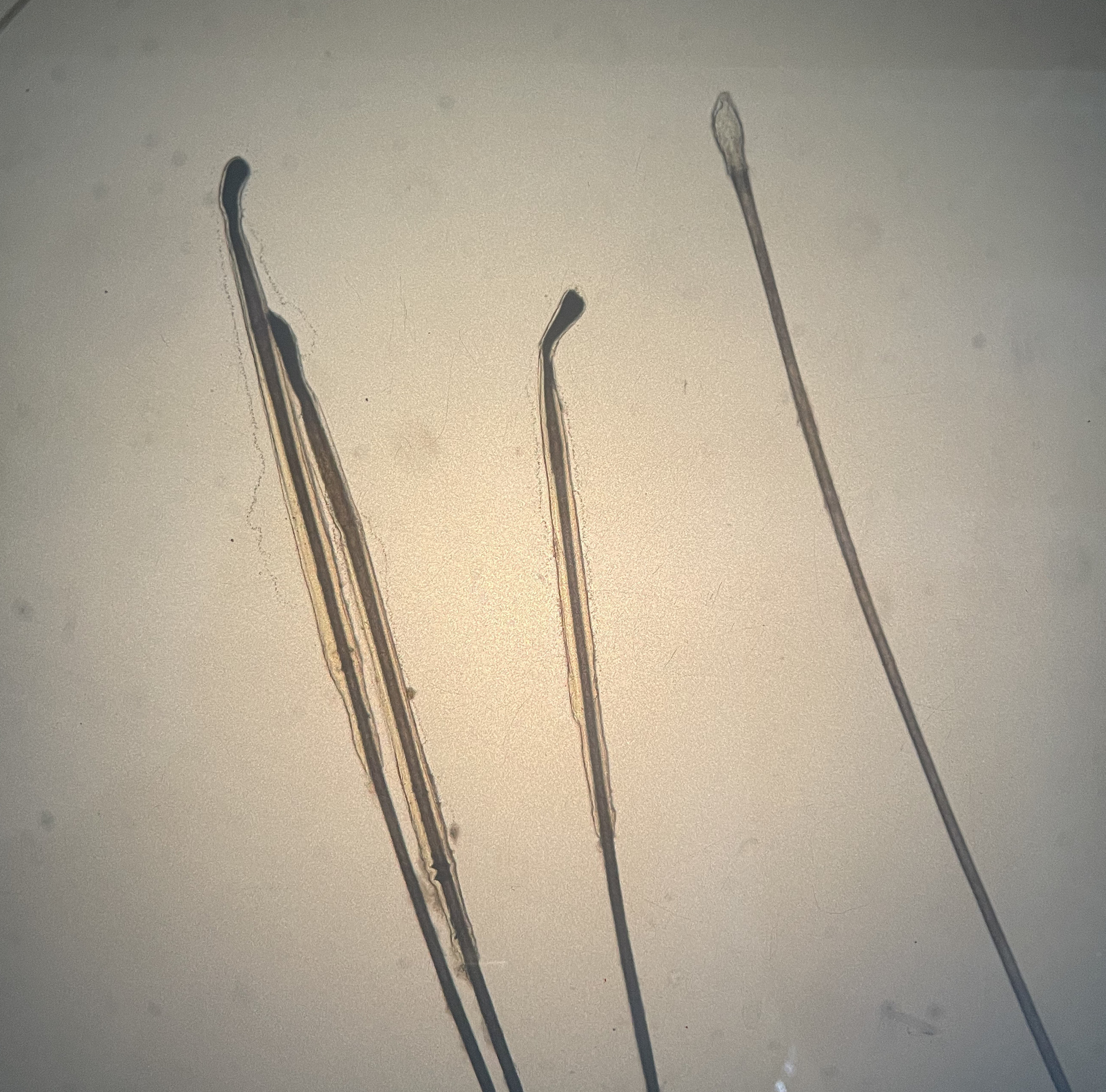

Supplement: Supplementary file 1 [file jcm-14-08471-s001.zip › Figure S1.jpg]
